# Prevalence and antimicrobial drug resistance of *Staphylococcus aureus* isolated from cow milk samples

**DOI:** 10.14202/vetworld.2020.2736-2742

**Published:** 2020-12-21

**Authors:** Matlale Phriskey Mphahlele, James Wabwire Oguttu, Inge-Marie Petzer, Daniel Nenene Qekwana

**Affiliations:** 1 Section Veterinary Public Health, Department of Paraclinical Sciences, Faculty of Veterinary Science, University of Pretoria, Pretoria, South Africa; 2 Department of Agriculture and Animal Health, College of Agriculture and Environmental Science, University of South Africa, Roodepoort, South Africa; 3 Department of Production Animal Studies, University of Pretoria, Pretoria, South Africa

**Keywords:** antimicrobial resistance, bovine mastitis, multidrug resistance, public health, *Staphylococcus aureus*

## Abstract

**Background and Aim:**

*Staphylococcus aureus* infections and antimicrobial resistance (AMR) in mastitis cases are both of clinical and economic importance. This study investigated the prevalence and AMR patterns of *S. aureus* isolated from composite milk samples of dairy cows submitted to the Onderstepoort Milk Laboratory for routine diagnosis.

**Materials and Methods:**

A total of 2862 cow milk samples randomly selected from submitted samples were tested for the presence of *S. aureus* using microbiological and biochemical tests. Confirmation of isolates was done using the analytical profile index. Antimicrobial susceptibility of *S. aureus* isolates against 12 antimicrobial agents was determined using the disk diffusion method.

**Results:**

*S. aureus* was isolated from 1.7% (50/2862) of the samples tested. All (100%) *S. aureus* isolates were resistant to at least one antimicrobial, while 62% (31/50) were resistant to three or more categories of antimicrobials (multidrug-resistant [MDR]). Most *S. aureus* isolates were resistant to erythromycin (62%; 31/50) and ampicillin (62%; 31/50). Almost half of *S. aureus* isolates were resistant to oxacillin (46%; 23/50) and only 8% (4/50) were resistant to cefoxitin.

**Conclusion:**

Although the prevalence of *S. aureus* among mastitis cases in this study was low, isolates exhibited high resistance to aminoglycosides, macrolides, and penicillins, all of which are important drugs in human medicine. The high prevalence of MDR *S. aureus* and the presence of methicillin resistance among *S. aureus* observed in this study are of both clinical and public health concerns.

## Introduction

*Staphylococcus* species are commensal of the skin and mucosal surfaces of animals and humans [1,2]. However, they have also been reported to cause clinical conditions such as food poisoning, toxic shock syndrome, and dermatitis in humans [3,4]. Among the coagulase-positive *Staphylococcus* species, the species predominantly associated with subclinical and clinical mastitis in dairy cattle is *Staphylococcus aureus* [5-7]. Staphylococcal mastitis cases are associated with decreased levels of milk production, increased levels of somatic cell count, and high rates of mastitis treatment failure. In addition, *S. aureus* udder infection is of economic significance as it often results in increased veterinary and treatment costs as well as premature culling of affected cows [8].

The treatment of choice for *S. aureus* mastitis includes antimicrobials such as β-lactams, tetracyclines, and aminoglycosides. On the other hand, glycopeptides, fluoroquinolones, and lincosamides are reserved for the treatment of methicillin-resistant *S. aureus* (MRSA) and multidrug-resistant (MDR) *Staphylococcus* species [6,9,10]. This notwithstanding, there are reports of β-lactams, aminoglycosides, and macrolides resistance among *S. aureus* [11,12].

This study investigated the burden of mastitis associated with *S. aureus* and the patterns of antimicrobial resistance (AMR) among *S. aureus* isolated from cow milk samples submitted to the Onderstepoort Milk Laboratory for routine clinical diagnosis. The study is premised on the hypothesis that the contribution of *S. aureus* to the overall burden of infectious mastitis in South Africa is low. In addition, the authors hypothesize that the prevalence of AMR and MDR *S. aureus* is higher than previously reported.

## Materials and Methods

### Ethical approval

This study was approved by the Animal Ethics Committee of the University of Pretoria, Faculty of Veterinary Science (Ethics Reference No. V121-16).

### Study area, population and period

The Onderstepoort Milk Laboratory receives both composite and quarter milk samples from dairy farms across South Africa for routine diagnosis of mastitis. In this study, milk samples of 2862 randomly selected individual cows between July 2016 and January 2017 were used.

### Sample type and collection method

Composite cow milk samples (approximately equal volumes of milk from the four quarters of the udder in one vial) collected aseptically from all individual cows in a herd by trained personnel before milking were used for this study. The samples were identified, packaged, and transported on ice packs to reach the Onderstepoort Milk Laboratory within 24-48h. The samples were maintained at an average temperature of below 4°C and were cultured immediately on arrival at the laboratory.

### Sampling strategy

A multistage sampling technique was adopted to identify the samples used in this study. The first stage involved sampling herds. The number of herds (*n*) to be sampled was determined based on an estimated prevalence of 6% (unpublished laboratory data). The following formula was used 
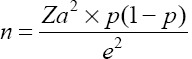
, where (*Za*) =95% confidence interval (CI) and e is the allowable error of 5% [13]. Therefore, 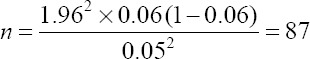. The number of herds was adjusted based on the formulae 
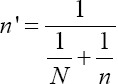
 [13], where *n* = original sample size estimate and *N* = size of the population. The total number of herds submitting milk samples to the laboratory per year was estimated to be 90. Therefore, over 6 months, the estimated number of herds to be sampled was calculated to be 45. After the adjustment, the total number of herds to be sampled for the duration of the study period was estimated to be 30. The second step was to calculate the number of animals to be sampled in each herd 25.1% herd prevalence [14]. The same formula described above was used: 
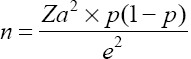
, 
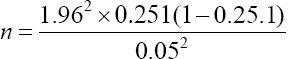
, *n* = 289. The number of animals to be sampled per herd was then adjusted using the formulae 
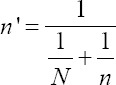
 as previously described.

### Isolation and identification of *S. aureus*

Using a sterile 10 μL plastic loop (Quality Biological, USA), milk samples were plated on blood tryptose agar and incubated at 37°C for 24 h. Presumptive *Staphylococcus* spp. colonies were initially identified based on phenotypic morphology, and biochemical tests as described by Quinn *et al*. [15] and the presence of coagulase using the slide agglutination test kit (Staphaurex^®^ kit, Oxoid, Thermo Fisher Scientific, USA). Coagulase-positive isolates were streaked onto a mannitol salt agar (Oxoid, Thermo Fisher Scientific, USA) and incubated for 24 h at 37°C [16]. Mannitol-positive isolates were confirmed to be *S*. *aureus* using the analytical profile index (API)^®^ Staph™ kit (API Staph test kit, Biomerieux, South Africa).

### Antimicrobial susceptibility tests

Mueller-Hinton agar (Oxoid, Thermo Fisher Scientific, USA) was used for antimicrobial susceptibility testing as described by the Clinical and Laboratory Standards Institute (CLSI) [17]. Isolates were subjected to a panel of 12 antimicrobial disks (Oxoid, Thermo Fisher Scientific, USA), which included ampicillin (AMP, 10 μg), erythromycin (E, 15 μg), chloramphenicol (C, 30 μg), linezolid (LZD, 30 μg), ciprofloxacin (CIP, 5 μg), vancomycin (VA, 30 μg), rifampicin (RD, 5 μg), trimethoprim/sulfamethoxazole (SXT, 25 μg), oxacillin (OX, 1 μg), polymyxin B (PB, 300 units), and cefoxitin (FOX, 30 μg). Although PB has low *in vitro* activity against Gram-positive bacteria, it is used in veterinary medicine for the treatment of *Staphylococcus* species dermatitis in combination with other antimicrobials. It is also suggested that at higher doses, it has effect against methicillin-resistant *S. aureus*. Furthermore, there is also evidence to suggest that miconazole and PB could be effective in the treatment of *Staphylococcus* infection [18-20]. One study showed that PB distributes well in the udder [21]. *Staphylococcus* *aureus* ATCC 25923 was used as a control. Results of the antimicrobial susceptibility tests were interpreted as prescribed in the CLSI guidelines [22]. However, the interpretation of the VA results was based on the criteria by Rezaeifar *et al*. [23]. For the purposes of this study, the intermediate-resistant isolates were reclassified as resistant. *S. aureus* isolates resistant to at least one antimicrobial drug were defined as AMR, while isolates that were resistant to three or more antimicrobial categories were classified as MDR [24].

### Statistical analysis

Crude percentages of *S. aureus*-positive samples and isolates that were AMR and MDR as well as their 95% CI were computed and presented as tables and figures. Statistical analysis was performed using SPSS v24.0 (IBM Corp., NY, USA).

## Results

Out of a total of 2862 milk samples that were tested, 1.7% (50/2862; CI: 1.3-2.3) were positive for *S. aureus*. All (100%, CI: 92-100) *S. aureus* isolates were AMR and 62% (31/50; CI: 81-97) of the isolates were MDR. *S. aureus* exhibited a high prevalence of resistance to PB (82%), E (62%), and AMP (62%). Low (8%) prevalence of resistance was observed against each of FOX, VA, and C. None of *S. aureus* isolates exhibited resistance to RD (Table-1).

**Table-1:** Distribution of antimicrobial resistance among *Staphylococcus aureus* isolated from milk samples submitted to the Onderstepoort Milk Laboratory.

Category	Drug	Number	Percent	95% CI^a^	
Polypeptides	Polymyxin B	41	82	69	90
Macrolides	Erythromycin	31	62	48	74
Penicillins	Ampicillin	31	62	48	74
	Oxacillin	23	46	33	60
Fluoroquinolones	Ciprofloxacin	10	20	11	33
Oxazolidinones	Linezolid	8	16	8	29
Sulfonamidees	Trimethoprim/sulfamethoxazole	5	10	4	21
Cephalosporins	Cefoxitin	4	8	3	19
Glycopeptides	Vancomycin	4	8	3	19
Phenicols	Chloramphenicol	4	8	3	19
Ansamycins	Rifampicin	0	0	0	7

a 95% confidence interval

**Figure-1:**
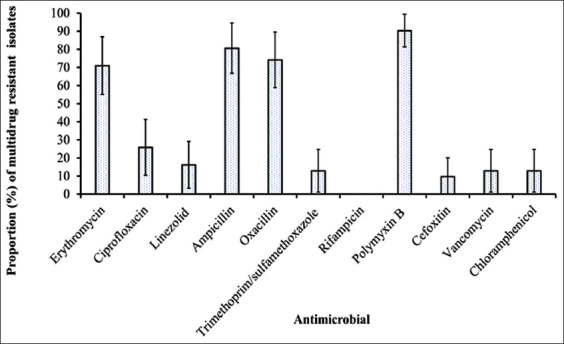
Proportions of multidrug-resistant *Staphylococcus aureus* that were resistant to each of the 11 antimicrobial agents.

Among *S. aureus* isolates that were MDR, majority were resistant to PB (83%), followed by E (66%), AMP (64%), and OX (49%) (Figure-1).

## Discussion

*S. aureus* infections and AMR in mastitis cases are both of clinical and economic importance [4,25]. In this study, a higher prevalence (1.7%; CI: 1.3-2.3) of *S. aureus* was observed as compared to 0.9% reported by Petzer *et al*. [26] in cow milk samples from dairy cattle in South Africa. However, the prevalence of *S. aureus* observed in this study is lower than 2.3% and 25.1% which was reported in earlier studies conducted on dairy cattle in South Africa by both Fosgate *et al*. [27] and Petzer *et al*. [14], respectively. Higher prevalence of *S. aureus* in cow milk samples was reported in Zimbabwe (16.3%) [28] and in Ethiopia (48.6%) [29]. Studies done in Sweden [30], Canada [31], and China [32] have also reported a higher proportion of *S. aureus* infection in cow milk samples, 21.3%, 21.7%, and 29.5%, respectively. The differences in the proportions of *S. aureus* in this study compared to what was observed in the other studies could be attributed to geographical differences, sampling methods, and study population. For example, Katsande *et al*. [28] used convenience sampling. In addition, the differences may also be due to different management practices, treatment, and control strategies [4]. Nonetheless, the low prevalence observed in this study suggests that *S. aureus* is not common among dairy cattle that were investigated in this study.

### AMR among *S. aureus* isolates from milk samples

All *S. aureus* isolates in this study were resistant to at least one antimicrobial agent. Our findings are similar to those reported by Asrat *et al*. [33] and Fikru [6] in Ethiopia as well as Schmidt [11] in KwaZulu Natal, South Africa, who reported 100% resistance to at least one antimicrobial. However, Mohammed [34] reported a slightly lower proportion (80.4%) of *S. aureus* resistant to at least one antimicrobial from dairy cows with mastitis in Tanzania. The results reported in the present and in other studies conducted in South Africa, further confirm the occurrence of high resistance among *S. aureus* from mastitis cases in dairy cattle. In light of this, there is a need to develop programs to promote prudent use of antimicrobial drugs to help curb or reduce the development of resistance among *S. aureus* from dairy cattle in South Africa [35].

### E and PB-resistant *S. aureus*

We observed a higher proportion of *S. aureus* resistant to E (62%) compared to 23.5% from cow milk samples reported in Ethiopia [33]. However, our findings are consistent with a prevalence of 69.2% reported by another study done in Ethiopia by Haftu *et al*. [36]. Although macrolides have been used for the treatment of mastitis in other countries, they are not routinely used for the treatment of mastitis in South Africa [37,38]. Therefore, it was not anticipated that such high levels of resistance against the macrolide, E, would be observed in this study. The reason for this observation is not clear. However, the authors are of the view that this could be due to cross-resistance with other antimicrobials commonly used in the dairy industry. This is supported by available evidence that suggests that resistance against macrolides that are caused by methylation of the ribosomal target of the antibiotics, leads to cross-resistance to macrolides, lincosamides, and streptogramins B, the so-called MLS_B_ phenotype [39]. The high proportion of resistance observed against PB (82%) was anticipated. This is because the antimicrobial lacks *in vitro* activity against Gram-positive organisms [40].

### β-Lactam-resistant *S. aureus*

With regard to resistance against β-lactams, our findings are in agreement with the findings of another South African study by Schmidt [11] that reported 65.6% prevalence of AMP resistance among *S. aureus* isolates. However, a higher proportion of resistance to AMP was observed in this study compared to studies done in South Africa that reported a prevalence of 28.8% [12], 54.5% in Egypt [41], and 57.0% in Kenya [42].

Studies conducted elsewhere, have reported a higher proportion of AMP-resistant *S. aureus* in dairy cattle compared to what we observed in this study. For example, Faris Beyene [43] reported a prevalence of 96%, while Haftu *et al*. [36] reported a prevalence of 82.4% in studies done in Ethiopia. Almost half (46%) of *S. aureus* isolates in this study were resistant to OX. This is similar to 52.9% resistance to the same drug that was reported in India [44]. On the contrary, we observed a higher prevalence of resistance to OX than 1.1% reported in an earlier study done in South Africa by Schmidt [11]. Similarly, our findings were higher than 29.7% reported by Byarugaba *et al*. [45] in Uganda and 33.3% reported by Asrat *et al*. [33] in Ethiopia. The high prevalence of AMP and OX resistance in this study is of great concern and could be because penicillins and other β-lactams are routinely used in the treatment of mastitis in South Africa. Therefore, intervention strategies are needed to curb the high prevalence of resistance to β-lactams observed in this study. However, the resistance levels observed against OX should be interpreted with caution given that OX disk diffusion testing is not reliable for detecting OX/methicillin resistance [46]. It is recommended that FOX be used instead as a surrogate for disk diffusion testing when investigating OX/methicillin resistance.

### FOX-resistant *S. aureus*

The *MecA* gene is the gold standard for detecting MRSA [47], and FOX is used as a proxy to test for the presence of *MecA* gene in MRSA strains [22]. While Schmidt *et al*. [12] in their study of *S. aureus* isolated from dairy cow milk samples in South Africa did not observe FOX resistance, the present study observed that 8.0% of *S. aureus* isolates were resistant to FOX. Nonetheless, this was lower than 32.4% reported by Ketema [48] and 67.2% reported by Tesfaye [49] from mastitis cases in Ethiopia. Although the results reported here suggest that the prevalence of MRSA was low, its presence is nonetheless a serious public health challenge given that MRSA is not only resistant to β-lactam antimicrobials [50] but also tends to be MDR [51] and is associated with poor prognosis [52]. In addition, dairy cattle can act as a source of MRSA infections for humans [12].

### Vancomycin-resistant *S. aureus* (VRSA)

VA is the drug of choice for the treatment of MRSA and MDR *S. aureus* infections [53,54]. In this study, the proportion of VRSA (8%) was higher than 2.2% reported in Tanzania [34] and 2.4% reported in Ethiopia [33]. In contrast, 16.0% resistance reported by Belayneh *et al*. [55], 52.4% by Daka *et al*. [56], and 38.5% by Bitewa [57] from milk samples of dairy cattle in Ethiopia were higher than what was observed in this study. Given that VA is not commonly used for the treatment of mastitis in South African dairy herds, the presence of VRSA in this study was not expected and is thus a grave public health concern. This warrants further research to determine the molecular characteristics of these isolates. Studies are also needed to investigate the cause of resistance observed against VA in this study. However, given that the disk diffusion test does not differentiate S. aureus isolates that are VA-susceptible from VA-intermediate strains, findings reported here may be an overestimation of the occurrence of VA resistance among *S. aureus* from the herds under study. Therefore, minimal inhibitory concentration test should be performed on isolates resistant to VA.

### MDR *S. aureus*

The presence of MDR *S. aureus* mastitis cases in dairy cattle has been reported extensively [6,58-60]. Therefore, the high proportion of MDR *S. aureus* observed in the present study was expected. Of concern though, is that the level of MDR *S. aureus* was higher than the 1.4% reported previously in South Africa [12]. Furthermore, the prevalence of MDR (62%) observed in this study was higher than 47.6% reported in Ethiopia [33], 26.1% in Tanzania [34], and 22.2% reported in Italy [58]. The high proportions of MDR *S. aureus* isolates observed in this study suggest that multidrug resistance is common among *S. aureus* from dairy herds that were investigated. Worth noting is that MDR *S. aureus* isolates tended to exhibit resistance mainly toward streptomycin and E. Although not observed in this study, other studies have also reported penicillin resistance as being common among MDR *S. aureus* isolates [33,61].

The present study is not without limitations. For example, this study investigated the prevalence and AMR of *S. aureus* among herds that submit milk to the Onderstepoort Milk Laboratory. Therefore, the results of this study cannot be generalized to all dairy herds in South Africa. In addition, herds included in this study are part of the herd health improvement program and hence are constantly monitored for conditions like mastitis. In view of this, it is possible that findings reported here could be an underestimation of the prevalence of *S. aureus* and AMR among dairy herds under study. Nonetheless, this study contributes to an improved understanding of the current burden and AMR patterns among dairy herds in South Africa.

## Conclusion

The prevalence of *S. aureus* among dairy herds in this study was low. *S. aureus* isolates tended to exhibit resistance mainly against aminoglycosides, macrolides, and penicillins. The high prevalence of MDR *S. aureus* and the possibility of MRSA due to resistance to OX and FOX observed in this study are of serious public health concern. The presence of VA resistance isolates warrants further molecular investigation to improve our understanding of the drivers of resistance against this antimicrobial.

## Authors’ Contributions

MPM, DNQ, and JWO designed the study. MPM and I-MP collected data and did laboratory analysis. MPM and DNQ analyzed the data. JWO, I-MP, and DNQ reviewed the manuscript. DNQ and JWO were the supervisors for the study. All authors read and approved the final manuscript.
